# Evaluation of Thermal Inactivation and Chemical Disinfection Efficacy Against Lassa Virus

**DOI:** 10.3390/v18040412

**Published:** 2026-03-27

**Authors:** Mengli Yang, Zhidan Zhang, Cong Cai, Kaiyun Ding, Xueping Chen, Shanhe Wu, Xin Guo, Qiangming Sun, Yunchuan Wang

**Affiliations:** National Kunming High-Level Biosafety Primate Research Center, Institute of Medical Biology, Chinese Academy of Medical Sciences & Peking Union Medical College, Kunming 650118, China; yangmengli7@163.com (M.Y.); zhidanzhang0414@163.com (Z.Z.); caicong@imbcams.com.cn (C.C.); dkydky_520@imbcams.com.cn (K.D.); cxp3100@imbcams.com.cn (X.C.); m15911746495_1@163.com (S.W.); 19988346414@163.com (X.G.)

**Keywords:** Lassa virus, thermal inactivation, disinfectant validation, BSL-4 laboratory, biosafety

## Abstract

Lassa virus (LASV), the causative agent of Lassa fever, must be handled under biosafety level 4 (BSL-4) conditions, requiring validated inactivation protocols to ensure laboratory and public safety. Although LASV is an enveloped virus theoretically susceptible to physical and chemical inactivation methods, quantitative data on its inactivation kinetics remain limited. This study systematically evaluated the efficacy of thermal treatment (56 °C, 70 °C, 95 °C), laboratory chemical inactivants (beta-propiolactone, formaldehyde, methanol, TRIzol), and five commercial disinfectants against infectious LASV. Viral infectivity was determined by titrating residual virus in Vero E6 cells, and complete inactivation was verified by three consecutive blind passages. Thermal inactivation was achieved at 56 °C for 40 min, 70 °C for 5 min, and 95 °C for 2 min. Both 0.1% and 0.05% beta-propiolactone completely inactivated LASV after 24 h at 4 °C, while 4% formaldehyde, 50% methanol, and 25% TRIzol achieved complete inactivation within 15 min, 10 min, and 2 min, respectively. For surface disinfection, 2% and 5% Micro-Chem Plus™ and 75% ethanol reduced viral titers by ≥4 log_10_ TCID_50_/mL within 30 s; 1% sodium hypochlorite and 0.25% Virkon required 1 min, whereas 3% hydrogen peroxide required 3 min to achieve the same reduction. These results provide quantitative, evidence-based parameters that can serve as a valuable reference for the safe handling of LASV under controlled BSL-4 laboratory conditions.

## 1. Introduction

Lassa virus (LASV), a member of the family *Arenaviridae*, is the causative agent of Lassa fever (LF), a severe acute viral hemorrhagic disease endemic to West Africa [1,2,3]. It is primarily transmitted to humans through contact with food or household items contaminated with the urine or feces of infected *Mastomys rodents* [4,5]. Human-to-human transmission can also occur via direct contact with the blood, secretions, or other body fluids of infected individuals, posing a particularly high risk in healthcare settings [6,7]. Although approximately 80% of infections are asymptomatic or mild, about 20% of cases progress to severe disease, affecting multiple organs such as the liver, spleen, and kidneys [8,9]. The case fatality rate among hospitalized patients with severe illness can exceed 15%, and it is even higher in pregnant women [10,11]. Due to its high pathogenicity and transmission risk, Lassa fever has been listed as a priority disease by the World Health Organization (WHO) [12]. Consequently, all work with live virus must be conducted within Biosafety Level 4 (BSL-4) laboratories.

Although LASV is an enveloped RNA virus theoretically susceptible to physical and chemical factors [13,14], previous studies have primarily focused on specific inactivation methods or conditions, often without systematic kinetic analysis. A recent comprehensive review by Nims and Ijaz [15] summarized the available data on physical and chemical inactivation of hemorrhagic fever viruses, including LASV, highlighting that while various microbicidal actives and formulations have demonstrated efficacy, quantitative data on systematic inactivation kinetics under controlled BSL-4 conditions remain limited. Current laboratory safety protocols and field disinfection guidelines often rely on extrapolation from related viruses or merely use nucleic acid detection as evidence of inactivation [16]. However, the latter may significantly underestimate the efficacy of the inactivation method, as many inactivation approaches can destroy viral infectivity while leaving nucleic acids intact, leading to false-positive detection signals. This lack of evidence-based critical data leaves the highest-level biosafety operations for LASV without a precise and reliable foundation, constituting a potential risk.

Therefore, to fill this gap, this study aimed to achieve two clear objectives through rigorous quantitative experiments: (1) to determine the thermal kinetics of LASV and evaluate the efficacy of laboratory chemical inactivants, providing definitive inactivation parameters for the deep and safe processing of infectious samples; and (2) to systematically evaluate the rapid virucidal efficacy of various commonly used environmental disinfectants, offering a scientific basis for selecting agents and timing for laboratory surface disinfection and outbreak response. All inactivation outcomes were validated using three consecutive blind passages as the gold standard, while disinfection efficacy was assessed by the log_10_ reduction value (LRV) in the quantitative suspension test. The parameters obtained under controlled BSL-4 laboratory conditions are expected to serve as a valuable reference for the development of evidence-based safety protocols.

## 2. Materials and Methods

### 2.1. Biosafety Statement

All experiments involving live LASV were performed in a nationally certified BSL-4 laboratory in strict compliance with the corresponding national biosafety regulations and standard operating procedures (SOPs) for BSL-4 facilities. All personnel involved in the experiments received rigorous BSL-4 training and personal protective equipment was worn in accordance with biosafety requirements throughout the study.

### 2.2. Cells and Virus

Vero E6 cells (ATCC CRL-1586) were cultured in Dulbecco’s Modified Eagle Medium (DMEM, Gibco, Waltham, MA, USA) supplemented with 10% fetal bovine serum (FBS, Gibco, Waltham, MA, USA), 100 U/mL penicillin and 100 μg/mL streptomycin. The cells were maintained in a humidified incubator at 37 °C with 5% CO_2_ and passaged when reaching 80–90% confluency.

The Lassa virus strain used in this study (designated LASV/China/2024/IMB-01) was isolated from a confirmed imported case in Sichuan, China, in 2024, by the Institute of Medical Biology, Chinese Academy of Medical Sciences, National Kunming High-Level Biosafety Primate Research Center, Yunnan, China [17]. Genomic analysis confirmed that the virus belongs to Lineage IV, and its complete genome sequences have been deposited in GenBase Database under accession numbers C_AA084677.1 (L segment) and C_AA084676.1 (S segment). The virus was passaged twice (P2) in Vero E6 cells to generate working stocks, and all experiments were conducted using virus from this passage level.

### 2.3. Virus Cultivation and Titer Determination

For LASV propagation, confluent Vero E6 cell monolayers in T225 cell culture flasks were inoculated with LASV at a multiplicity of infection (MOI) of 0.5. After 1 h of adsorption at 37 °C, the inoculum was removed and fresh complete DMEM was added. The infected cells were incubated at 37 °C with 5% CO_2_ for 5 days, after which the cell culture supernatant was collected. The remaining cell monolayer was subjected to three freeze–thaw cycles to release intracellular virus, and the lysate was combined with the collected supernatant. The mixed viral suspension was centrifuged at 3000× *g* for 5 min to remove cell debris, and the clarified supernatant was aliquoted and stored at −80 °C as viral stock for subsequent experiments.

Viral titer was determined using the Spearman–Kärber method [18] and expressed as log_10_ 50% cell culture infective dose per milliliter (log_10_ TCID_50_/mL). Briefly, serial 10-fold dilutions of the viral suspension were prepared in complete DMEM, and 100 μL of each dilution was inoculated into quadruplicate wells of 96-well plates containing 2–3 × 10^4^ Vero E6 cells per well. After 5 days of incubation at 37 °C with 5% CO_2_, the cytopathic effect (CPE) was observed under an inverted light microscope. The lower limit of quantification (LLOQ) of this titration method was 1.5 log_10_ TCID_50_/mL.

### 2.4. Thermal Inactivation Experiment

Aliquots of 0.5 mL LASV stock (initial titer: 8.29 log_10_ TCID_50_/mL) were dispensed into sterile microcentrifuge tubes and subjected to thermal treatment in a digital metal bath at 56 °C, 70 °C or 95 °C for predetermined durations (56 °C: 10, 20, 30, 40, 50, 60, 70, 90 min; 70 °C and 95 °C: 2, 5, 10, 15, 20 min). Immediately after the designated exposure time, the tubes were transferred to an ice bath to rapidly terminate the thermal inactivation reaction. The residual viral titer in each treated sample was then determined using the method described in Section 2.3. Untreated viral stock was used as the positive control.

### 2.5. Evaluation of Chemical Treatment Efficacy

#### 2.5.1. Determination of Cytotoxicity

Nine disinfectants were tested in this study: beta-propiolactone (BPL) (Serva, Heidelberg, Germany), formaldehyde (Servicebio, Wuhan, China), methanol (Xilong Scientific, Guangzhou, China), TRIzol (Invitrogen, Carlsbad, CA, USA), Micro-Chem Plus™ (MCP) (MCP, Carlsbad, CA, USA), 75% ethanol (Tiangen, Kunming, China), 1% sodium hypochlorite (NaClO) (Tiangen, Kunming, China), 3% hydrogen peroxide (H_2_O_2_) (Laiwo, Kunming, China), and potassium peroxymonosulfate complex (Virkon) (Sulfolk, UK).

To minimize cytotoxicity and terminate the disinfection reaction, all virus-disinfectant mixtures were diluted with ice-cold complete DMEM (2% FBS) immediately after the designated contact time [19,20]. Prior to testing, the cytotoxic effect of each diluted disinfectant was evaluated on Vero E6 cells. Briefly, test samples were serially diluted in complete DMEM (2% FBS), and 100 μL of each dilution was added to cell monolayers. Following 1 h of incubation at 37 °C, the dilutions were removed, cells were replenished with fresh culture medium, and incubated for 4 days. Cytotoxicity was assessed daily by light microscopy.

#### 2.5.2. Chemical Inactivation

To identify chemical reagents suitable for the deep inactivation of infectious LASV samples (for downstream applications such as nucleic acid extraction, serological testing and vaccine development), the following were tested: BPL, at final concentrations 0.1% and 0.05%, acting at 4 °C for 24 h followed by hydrolysis at 37 °C; For 4% formaldehyde, 50% methanol and 25% TRIzol treatment. The viral suspension was mixed with the inactivant at the specified concentration and incubated at room temperature for predetermined durations (formaldehyde: 15, 30, 60 min; methanol: 10, 30, 60 min; TRIzol: 2, 5, 10 min). The primary endpoint of this assay was the complete elimination of viral infectivity, which was verified by three consecutive blind cell passages (Section 2.6). Viral suspension without chemical treatment was used as the positive control, and complete medium containing the chemical inactivant (without virus) served as the negative control.

#### 2.5.3. Virucidal Efficacy of Environmental Disinfectants

To evaluate reagents for rapid disinfection of laboratory environments and equipment surfaces, the following were selected: 2% and 5% MCP, 75% ethanol, 1% sodium hypochlorite, 3% hydrogen peroxide, and 0.25% Virkon. The virucidal efficacy of the disinfectants was evaluated using a quantitative suspension test in accordance with international standard protocols. Briefly, LASV suspension and disinfectant solution were mixed at a volume ratio of 1:1 (*v*/*v*) at room temperature to achieve the desired working concentration of the disinfectant, and the mixture was incubated for predetermined exposure times (30 s, 1, 3, 5 min/10 min). Immediately after the designated exposure time, a 10 μL aliquot of the reaction mixture was removed and rapidly diluted with ice-cold complete DMEM to achieve the minimum dilution required to eliminate cytotoxicity for each disinfectant, as determined by prior cytotoxicity testing (Section 2.5.1). The dilution factors varied by disinfectant: 2% and 5% MCP, 3% H_2_O_2_, and 0.25% Virkon were diluted 1000-fold; whereas 1% NaClO and 75% ethanol were diluted 10-fold. The residual viral titer in the diluted sample was then determined, and the LRV was calculated to evaluate the disinfection efficacy. Each experiment was performed in triplicate.

### 2.6. Validation of Complete Inactivation by Three Consecutive Blind Passages

To confirm the absence of residual infectious virus in samples treated with chemical inactivants or thermal treatment (with titers below the LLOQ), all such samples were subjected to three consecutive blind passages in Vero E6 cells. Briefly, 100 μL of the treated sample was inoculated into confluent Vero E6 cell monolayers in 96-well plates and incubated at 37 °C with 5% CO_2_ for 5 days. The culture supernatant was then collected and inoculated onto fresh Vero E6 cells for the next passage, regardless of whether CPE was observed. This process was repeated for a total of three passages. If no CPE was observed in all three passages, and the positive control (untreated virus) exhibited typical CPE, the sample was judged to be completely inactivated. If CPE was observed in any passage, the sample was considered to contain residual infectious virus.

### 2.7. Data Analysis

All experimental data are presented as the mean ± standard deviation (SD) of at least three independent replicates. The LRV for each disinfectant was calculated using the formula: LRV = log_10_ (viral titer of the untreated control) − log_10_ (viral titer of the disinfectant-treated sample). In accordance with international standards for virucidal activity testing [21], a disinfectant was considered effective if it achieved an LRV ≥ 4 at a given contact time. As the primary objective of this study was to determine whether each tested condition met this predefined efficacy threshold, results are presented descriptively, and no further statistical comparisons were performed.

### 2.8. Kinetic Analysis of Thermal Inactivation

To quantify the thermal stability of LASV, the time-dependent reduction in viral titers at 56 °C was fitted to a first-order kinetics model. The decimal reduction time (*D*-value), defined as the time required to reduce the virus titer by 90% (1 log_10_) at a given temperature, was calculated from the negative reciprocal of the slope obtained by linear regression analysis of log_10_-transformed titers versus time. Only data points above the lower limit of quantification (LLOQ, 1.5 log_10_ TCID_50_/mL) were included in the regression. For 70 °C and 95 °C, where rapid inactivation limited the number of quantifiable time points, *D*-values were estimated conservatively based on the reduction observed at the earliest time point or the detection limit, as described in the Section 3.

## 3. Results

### 3.1. Thermal Inactivation Kinetics

To clarify the key parameters for thermal inactivation of LASV, this study systematically measured the residual infectivity of the virus after treatment at 56 °C, 70 °C, and 95 °C for different durations. The initial titer of the virus stock used was 8.29 log_10_ TCID_50_/mL.

As shown in Table 1, the inactivation rate of LASV exhibited significant temperature dependence. At 56 °C, the virus titer decreased in a time-dependent manner: after 30 min of treatment, the average titer dropped to 2.25 ± 0.25 log_10_ TCID_50_/mL. Critically, after 40 min, the virus titer fell below the LLOQ (<1.5 log_10_ TCID_50_/mL) and was confirmed as completely inactivated by three consecutive blind passages showing no CPE, corresponding to a log_10_ reduction of >6.79. This 40 min time point represents the first duration at which complete inactivation was achieved at 56 °C.

When the temperature was increased to 70 °C, inactivation efficiency improved substantially. After 2 min of treatment, the average titer dropped to 3.50 ± 0.25 log_10_ TCID_50_/mL, whereas after 5 min of treatment, the virus titer dropped below the LLOQ and successfully passed three blind passages, indicating complete inactivation.

**Figure 1:**
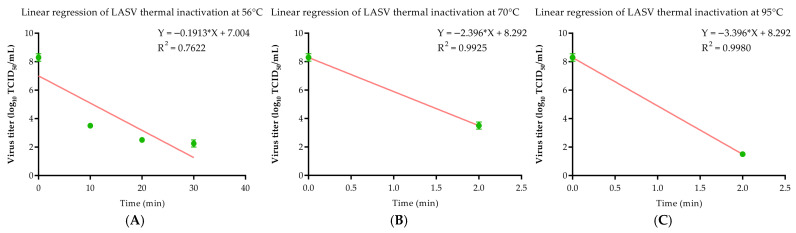
Thermal inactivation kinetics of LASV. Virus stocks (8.29 log_10_ TCID_50_/mL) were heated at indicated temperatures. Residual infectivity was titrated in Vero E6 cells. Data are mean ± SD (*n* = 3); green dots represent individual data points; Red line: linear regression fit. (**A**) 56 °C. Linear regression of data at 0–30 min yielded the equation: log_10_(titer) = 7.004 − 0.1913 × time (R^2^ = 0.762), corresponding to a *D*-value of 5.23 min. (**B**) 70 °C. The titer dropped to 3.5 log_10_ TCID_50_/mL within 2 min (estimated *D* < 0.42 min). (**C**) 95 °C. Complete inactivation (>6.79 log_10_ reduction) occurred within 2 min (estimated *D* < 0.3 min).

To further characterize the thermal stability of LASV, kinetic analysis was performed on the timeseries data (Figure 1A–C). At 56 °C, linear regression of log_10_–transformed titers at 0, 10, 20, and 30 min yielded a *D*-value of 5.227 min (R^2^ = 0.7622), confirming that inactivation follows first-order kinetics at this temperature. At 70 °C, the virus titer decreased from 8.29 to 3.5 log_10_ TCID_50_/mL within 2 min, corresponding to a reduction of 4.79 log_10_. Based on this single quantifiable time point, the *D*-value was estimated to be <0.417 min (<25 s), assuming first–order decay. At 95 °C, the virus was inactivated to below the LLOQ within 2 min, achieving a >6.79 log_10_ reduction, which gives a estimated *D*-value of <0.294 min (<18 s). For 70 °C and 95 °C, precise *D*-values could not be calculated due to the limited number of quantifiable time points resulting from rapid inactivation; therefore, the reported values should be considered as conservative estimates. These kinetic parameters are summarized in Table 1 (*D*-value and R^2^ columns) and provide a quantitative basis for comparing LASV thermal stability with other enveloped viruses.

### 3.2. Virucidal Efficacy of Chemical Treatment Efficacy

#### 3.2.1. Cytotoxicity Verification and Reaction Termination Protocol

To ensure the accuracy of subsequent virus detection, the cytotoxicity of each disinfectant after serial dilution was first assessed. As shown in Table 2, all tested disinfectants at their working concentrations, 0.1% BPL, 4% Formaldehyde, 50% methanol when diluted 10,000 fold, had no toxic effect on Vero E6 cells. 2% and 5% MCP, 3% H_2_O_2_, 25% TRIzol, 0.25% Virkon, when diluted 1000 fold, had no toxic effect on Vero E6 cells. 1% NaClO and 75%Ethanol had no toxic effect on Vero E6 cells.

Therefore, in this study, posttreatment dilution at different fold ratios was adopted as a uniform and reliable method to terminate the reaction and enable detection.

#### 3.2.2. Complete Inactivation by Laboratory Chemical Inactivants

This section evaluated chemical reagents for deep inactivation of laboratory samples (e.g., for inactivated vaccine preparation, serological testing samples) or pre-treatment for nucleic acid extraction. The goal was to ensure no residual live virus in the samples; therefore, “no CPE after three consecutive blind passages” was used as the gold standard for complete inactivation.

As shown in Table 3, BPL at a final concentration of 0.05% treated at 4 °C for 24 h completely inactivated LASV.

For the traditional agents tested, formaldehyde (4%) exhibited complete inactivation at all time points evaluated (15, 30, and 60 min). Similarly, methanol (50%) showed complete inactivation at the tested exposures of 10, 30, and 60 min. In contrast, the nucleic-acid extraction reagent TRIzol (25%) acted extremely rapidly, achieving complete inactivation within 2 min.

### 3.3. Disinfectant Inactivation Efficacy

This section evaluated reagents for rapid disinfection of environmental surfaces, instruments, or hands, using the internationally recognized quantitative suspension test standard with a LRV ≥ 4 as the criterion for effective disinfection [22].

After confirming no cytotoxicity interference, the virucidal efficacy of five categories of commonly used disinfectants against LASV was systematically evaluated using the quantitative suspension test (Table 4). All disinfectants exhibited effective virucidal activity (≥4 log_10_ reduction) at their recommended working concentrations.

For rapid disinfection, 2% and 5% MCP, 75% ethanol, 1% sodium hypochlorite, and 0.25% Virkon all achieved complete inactivation (LRV ≥ 4) within just 30 s of contact with the virus, demonstrating extremely rapid onset. These agents are particularly suitable for scenarios requiring immediate response, such as laboratory spills. By comparison, 3% hydrogen peroxide required 3 min of contact to achieve complete inactivation (LRV ≥ 4). At shorter exposure times of 30 s and 1 min, the LRV was approximately 2.38 and 3.38, respectively, indicating a time-dependent efficacy profile [23]. This finding underscores the critical importance of adhering to manufacturer-recommended or validated contact times for reliable disinfection outcomes.

## 4. Discussion

In the present study, we conducted a comprehensive and systematic evaluation of the thermal inactivation and chemical disinfection efficacy against LASV, a highly pathogenic BSL-4 pathogen, using rigorous quantitative experimental methods and the gold standard of blind cell passage for validating complete viral inactivation. This study establishes a comprehensive database of inactivation and disinfection parameters for LASV, covering physical thermal treatment, laboratory chemical inactivants and commercial environmental disinfectants. The results clearly distinguish two technical pathways for LASV inactivation suitable for sample processing and rapid environmental disinfection, respectively, and provide direct experimental evidence and laboratory-based guidance for the safe handling of LASV under controlled BSL-4 conditions and for informing emergency response protocols.

A broader survey of viral heat inactivation reveals substantial inter-family variability in thermostability among enveloped viruses. Nims and Plavsic [24] compiled data from multiple virus families and calculated the temperature required to achieve a 1 log_10_ reduction in 30 s. Their analysis showed that enveloped virus families display a wide range of thermostability. For instance, viruses in the *Rhabdoviridae* and *Orthomyxoviridae* families required approximately 59 °C to achieve a 1 log_10_ reduction in 30 s, whereas viruses in the *Coronaviridae* and Herpesviridae families required higher temperatures, around 76–79 °C. Our finding that LASV requires 40 min at 56 °C for complete inactivation suggests that its thermostability may be positioned at the higher end of the spectrum for enveloped viruses. This inter-family variability underscores the critical importance of generating empirical, virus-specific inactivation data, as demonstrated in the current study for LASV, rather than relying on data from related or surrogate viruses. The effectiveness of all heat treatment conditions was rigorously validated by three consecutive blind passages—a step crucial for ensuring the absolute safety of samples leaving the BSL-4 core area [25,26].

Regarding chemical treatments, this study clarified two parameter systems for different purposes. For sample pre-treatment aimed at permanently eliminating viral infectivity for downstream analyses (e.g., nucleic acid testing, serological testing), we evaluated deep inactivants. The result that BPL completely inactivated LASV at both 0.1% and 0.05% concentrations underscores that even the lower concentration is sufficient under the tested conditions (24 h at 4 °C). This provides a validated and potentially more practical option for safe production in fields such as vaccine development. Formaldehyde (4%) exhibited complete inactivation at 15, 30, and 60 min, while methanol (50%) showed the same efficacy at 10, 30, and 60 min. TRIzol (25%) provided near-instantaneous inactivation within 2 min. These defined parameters provide clear and safe options for sample pre-processing in different fields such as pathological examination and genomics.

Our findings for chemical inactivation are generally consistent with previous reports. Using a similar suspension test approach (ASTM E1052-20), Cutts et al. [27] demonstrated that 0.5% sodium hypochlorite and 67% ethanol achieved complete inactivation of LASV within 1–5 min under organic load conditions (tripartite soil containing bovine serum albumin, tryptone, and mucin), whereas our study was conducted under clean conditions. This likely explains the faster inactivation observed in our study (≥4 log_10_ reduction within 30 s for 1% sodium hypochlorite and 75% ethanol), as the presence of organic load can interfere with disinfectant efficacy. Similarly, Olejnik et al. [26] reported that TRIzol and aldehyde-based fixatives reliably inactivate LASV, which is consistent with our findings that 25% TRIzol achieved complete inactivation within 2 min and 4% formaldehyde within 15 min. These comparisons highlight the importance of considering experimental parameters when interpreting inactivation data for biosafety protocol development.

On the other hand, for routine laboratory disinfection within controlled BSL-4 facilities, this study confirmed the rapid effectiveness of multiple commonly used disinfectants under standardized suspension test conditions. It is worth noting that 2% and 5% MCP, 75% ethanol, 1% sodium hypochlorite, and 0.25% Virkon all achieved complete inactivation (LRV ≥ 4) within 30 s of contact. These findings provide a quantitative basis for the selection of rapid-response agents for laboratory work surfaces, equipment, and hands in the event of spills or accidents under controlled conditions [28,29,30]. Sodium hypochlorite, Virkon and hydrogen peroxide were also effective. However, the data showing that 3% hydrogen peroxide required 3 min of contact time to achieve complete effect (LRV ≥ 4), while achieving only partial reduction at shorter times (LRV ~1.38 at 30 s, ~2.63 at 1 min), provides critical, quantitative support for emphasizing “sufficient contact time” when formulating disinfectant SOPs. These data, based on the quantitative suspension test, establish a reliable baseline for evaluating the intrinsic virucidal efficacy of disinfectants under controlled laboratory conditions.

The methodological strength of this study lies in its multi-level, high-rigor validation strategy. We adhered to cell infectivity (quantifying infectious virus titers as TCID_50_) as the gold standard for evaluation, avoiding the potential misjudgment of residual risk associated with relying solely on nucleic acid detection. Particularly crucial was the adoption of three consecutive blind passages—the most stringent biosafety validation standard—for complete inactivation. For disinfectant evaluation, we established a uniform physical dilution termination method through systematic cytotoxicity pre-testing, ensuring comparability and accuracy of results. These methodological considerations collectively enhance the reliability and reference value of the entire study. This study employed suspension tests to evaluate disinfectant efficacy, which may raise the question of why this approach was chosen for agents intended for surface disinfection. Suspension tests are internationally recognized standard methods (e.g., EN 14476 [21], ASTM E1052-20 [31]) for initial screening of microbicidal activity, as they provide a standardized, reproducible, and quantitative assessment of a disinfectant’s intrinsic virucidal efficacy against a target virus. In the context of BSL-4 containment, suspension tests also offer a practical and safer approach for comparing multiple disinfectants under controlled conditions before progressing to more complex carrier tests. However, we acknowledge that this approach has limitations. All inactivation experiments were conducted using clean virus suspensions, which represent an idealized scenario and may not fully reflect real-world contamination scenarios where organic materials (e.g., blood, serum, or respiratory secretions) are present, and where viruses may be dried on surfaces. Therefore, the inactivation parameters reported here should be interpreted as baseline efficacy under optimal conditions. In practice, the presence of organic load could reduce disinfectant efficacy, and users should consider this when applying these results to develop safety protocols. Future research employing carrier tests with organic loads is warranted to validate these findings for practical surface disinfection applications [32,33].

In summary, this study systematically quantified the key inactivation and disinfection parameters for LASV under controlled BSL-4 laboratory conditions and provided differentiated evaluation criteria based on different biosafety application scenarios (sample processing vs. rapid environmental disinfection). These empirical data can serve as a valuable reference for the development and refinement of biosafety operating procedures to guide risk management within BSL-4 laboratories, and provide a scientific basis for informing public health departments in their efforts to establish evidence-based disinfection guidelines for Lassa virus. It is important to note that these findings are derived from suspension tests under clean conditions and should not be directly extrapolated to real-world disinfection scenarios without further validation using carrier tests or organic load conditions.

## Figures and Tables

**Table 1:** Thermal inactivation of LASV at different temperatures. Under the extreme high temperature of 95 °C, inactivation was extremely rapid. Just 2 min of treatment reduced the virus titer below the LLOQ, and three blind passages yielded negative results, achieving reliable and thorough inactivation.

Temp	Time	Virus Titer log_10_ TCID_50_/mL; Mean + SD		CPE (1/2/3Passage)	Inactivated (Y/N)	*D*-Value (min)	R^2^
		Pre-Heating	After-Heating				
(A)							
56 °C	10 min	8.29 ± 0.21	3.5 ± 0	+/+/+	N	5.227	0.7622
	20 min		2.5 ± 0	+/+/+	N		
	30 min		2.25 ± 0.25	+/+/+	N		
	40 min		ND	−/−/−	Y		
	50 min		ND	−/−/−	Y		
	60 min		ND	−/−/−	Y		
	70 min		ND	−/−/−	Y		
	90 min		ND	−/−/−	Y		
(B)							
70 °C	2 min	8.29 ± 0.21	3.50 ± 0.25	+/+/+	N	<0.417 (25 s) *	N/A
	5 min		ND	−/−/−	Y		
	10 min		ND	−/−/−	Y		
	15 min		ND	−/−/−	Y		
	20 min		ND	−/−/−	Y		
(C)							
95 °C	2 min	8.29 ± 0.21	ND	−/−/−	Y	<0.294 (<18 s) ^†^	N/A
	5 min		ND	−/−/−	Y		
	10 min		ND	−/−/−	Y		
	15 min		ND	−/−/−	Y		
	20 min		ND	−/−/−	Y		

Note: ND, not detected (below the lower limit of quantification [LLOQ] of 1.5 log_10_ TCID_50_/mL). Initial titer: 8.29 log_10_ TCID_50_/mL. CPE: cytopathic effect in three blind passages; “+” indicates CPE observed, “−” indicates no CPE. Y, completely inactivated; N, not completely inactivated. *D*-value: time required to reduce virus titer by 90% (1 log_10_). For 56 °C, *D*-value (5.227 min) was calculated by linear regression of titers at 0–30 min (R^2^ = 0.7622). * Estimated from reduction from 8.29 to 3.5 log_10_ TCID_50_/mL within 2 min at 70 °C (<0.417 min/25 s). ^†^ Estimated estimate based on complete inactivation (>6.79 log_10_ reduction) within 2 min at 95 °C (<0.294 min/18 s). R^2^ not applicable (N/A) for 70 °C and 95 °C due to insufficient quantifiable data points.

**Table 2:** Results of cytotoxicity pre–test for disinfectants.

Tested Agents	Work Concentration (*v*/*v*)	Dilution (of Tested Conc.)			
		10^1^	10^2^	10^3^	10^4^
BPL	0.1%	+	+	+	−
Formaldehyde	4%	+	+	+	−
Methanol	50%	+	+	+	−
TRIzol	25%	+	+	−	−
MCP	5%	+	+	−	−
MCP	2%	+	+	−	−
H_2_O_2_	3%	+	+	−	−
Virkon	0.25%	+	+	−	−
NaClO	1%	−	−	−	−
Ethanol	75%	−	−	−	−

Note: Cytotoxicity was assessed in three independent experiments, each with 4 replicate wells. “+” indicates cytotoxicity (cell lysis/death) observed in all replicates at that dilution; “−” indicates no cytotoxicity observed in any replicate. All three experiments gave identical results for each condition.

**Table 3:** Chemical inactivation by BPL and other agents.

Inactivant	Concentratin (*v*/*v*)	Time	CPE (1/2/3Passage)	Inactivated (Y/N)
BPL	0.1%	24 h	−/−/−	Y
	0.05%		−/−/−	Y
Formaldehyde	4%	15 min	−/−/−	Y
		30 min	−/−/−	Y
		60 min	−/−/−	Y
Methanol	50%	10 min	−/−/−	Y
		30 min	−/−/−	Y
		60 min	−/−/−	Y
TRIzol	25%	2 min	−/−/−	Y
		5 min	−/−/−	Y
		10 min	−/−/−	Y

Note: “−”, no cytopathic effect observed; “Y”, yes.

**Table 4:** Virucidal efficacy of common environmental disinfectants against LASV.

Disinfectant	Concentration (*v*/*v*)	Time	Virus Titer log_10_ TCID_50_/mL			LRV
			Undisinfected	Post-Dilution	Disinfected	
MCP	5%	30 s	8.29 ± 0.21	5.63 ± 0.35	ND	>4.13
		1 min			ND	>4.13
		3 min			ND	>4.13
		5 min			ND	>4.13
MCP	2%	30 s		5.63 ± 0.35	ND	>4.13
		1 min			ND	>4.13
		3 min			ND	>4.13
		5 min			ND	>4.13
Ethanol	75%	30 s		7.29 ± 0.16	ND	>5.79
		1 min			ND	>5.79
		3 min			ND	>5.79
		5 min			ND	>5.79
Virkon	0.25%	30 s		5.63 ± 0.35	ND	>4.13
		1 min			ND	>4.13
		3 min			ND	>4.13
		5 min			ND	>4.13
NaClO	1%	30 s		7.29 ± 0.16	ND	>5.79
		1 min			ND	>5.79
		3 min			ND	>5.79
		5 min			ND	>5.79
H_2_O_2_	3%	30 s		5.63 ± 0.35	3.25 ± 0.25	2.38 ± 0.43
		1 min			2.25 ± 0.25	3.38 ± 0.43
		3 min			ND	>4.13
		5 min			ND	>4.13

Note: The criterion for effective inactivation is an LRV ≥ 4. The LRV was calculated as the difference between the virus titers of the untreated control and the disinfectant-treated sample, both of which were processed under identical dilution conditions. ND: not detected.

## Data Availability

Data is contained within the article.
